# Modest Increase in Fertility Consultations in Female Adolescents and Young Adults with Lymphoma: A Population-Based Study

**DOI:** 10.1089/jayao.2020.0101

**Published:** 2021-06-15

**Authors:** Charlotte E.M. Coleman, Jessica Pudwell, Chad McClintock, Ann Korkidakis, Michael Green, Maria P. Velez

**Affiliations:** ^1^ Undergraduate Medicine, Queen's University, Kingston, Ontario, Canada.; ^2^ Department of Obstetrics and Gynecology, Queen's University, Kingston, Ontario, Canada.; ^3^ ICES, Queen's University, Kingston, Ontario, Canada.; ^4^ Department of Obstetrics and Gynecology, University of British Columbia, Vancouver, British Columbia, Canada.; ^5^ Department of Family Medicine, Queen's University, Kingston, Ontario, Canada.

**Keywords:** lymphoma, fertility preservation, epidemiology

## Abstract

While survival after hematological malignancies in adolescent and young adult patients is improving, patients report poor oncofertility care. This population-based, retrospective, cohort study used data from the Ontario Cancer Registry and billing codes to identify fertility consultations for lymphoma patients between 2000 and 2018. Consultation trends across time and different patient and physician characteristics were analyzed. We identified 2088 patients and a consultation rate of 3.4% (increasing from 1% in 2000–2006 to 8% in 2014–2018). Patient parity and regional deprivation scores decreased rates. Despite mild improvement, there is ample missed opportunity for fertility discussions.

## Introduction

The adolescent and young adult (AYA) age group, 15–39 years of age, presents unique challenges in oncology. Hematological malignancies (HMs) make up 21%–34% of cancer diagnoses among this age group. Since the 5-year survival rate for HMs diagnosed before the age of 39 ranges from 50% to 95%, research foci are shifting to improve quality of life (QOL) after cancer.^1^ An influencer of QOL is fertility, which can be affected by oncologic diagnoses and treatments. Greaves et al. demonstrated that HM survivors were more likely to remain childless than the general population.^2^ In Hodgkin's lymphoma (HL), the most common HM in the AYA population, premature ovarian failure occurs in up to 37% of patients.^3^ To address these concerns, the discipline of oncofertility emerged with the hopes to accelerate the inclusion of fertility in oncology care.^4^

The American Society of Clinical Oncology (ASCO) has released guidelines necessitating fertility discussions in oncology care for AYA patients.^5–7^ However, oncology patients continue to report dissatisfaction.^8^ Specifically, in AYA patients with HM, rates of infertility discussions vary from 17% to 83%.^2^ Factors that affect these rates include patient demographics, physician characteristics, and complex societal factors.^8,9^

While the need for fertility consultations led to the ASCO guideline updates, the impact of these guidelines is unknown. Similarly, patient surveys cannot capture the scope of care in a population. Our group published a population-based analysis of referral rates for AYA, breast cancer patients showing a modest improvement after the 2013 guideline.^10^ This article investigates a similar trend in lymphoma patients by capturing the province of Ontario. We determined patient, physician, and socioeconomic factors associated with fertility consultation rates, highlighting areas for improvement.

## Methods

This population-based cohort study of AYAs diagnosed with lymphoma from January 2000 to March 2018 identified AYAs residing in Ontario, Canada's largest province by population (13.2 million) using the Ontario Cancer Registry (>98% of cancer incidents).^11^ Patients with a history of infertility, sterilization procedures, or previous cancer and patients ineligible for health insurance were excluded.

Datasets were linked by encoded identifiers and analyzed at ICES (www.ices.on.ca). The primary outcome, gynecology consultation about fertility, between the diagnosis of HM and commencement of chemotherapy, was identified as a gynecology consult billed as an International Classification of Diseases, Ninth Revision (ICD-9) code 628 (infertility diagnosis).

Patient and physician demographics were retrieved from the Registered Persons Database and ICES Physician Database, respectively. Parity was defined as a previous live birth (MOMBABY dataset). Chemotherapy billing codes identified treatment commencement. Community deprivation and income quartile scores measured socioeconomic factors.

Individual characteristics were analyzed using chi-square analyses for categorical variables, one-way analysis of variance (ANOVA) for continuous variables, and Kruskal–Wallis tests for medians. Logistic regression examined how factors influenced fertility consults, adjusting for confounding variables through a backward selection modeling. All statistical tests were two sided with *p* < 0.05 for significance. Data were analyzed using SAS version 9.4 (Cary, NC). This study was approved by the Queen's University Health Sciences and Affiliated Teaching Hospitals Research Ethics Board.

## Results and Discussion

This population-based study assessed fertility consultation rates in female AYAs with lymphoma in Ontario, Canada. A total of 2088 female AYAs diagnosed with lymphoma were identified. Table 1 presents sociodemographic characteristics with the adjusted model in Table 2. HL represented 1323 (63%) of the study population, whereas, 765 (37%) had non-Hodgkin's lymphoma (NHL). The overall referral rate (3.4%) was low, ranging from below 1% before 2006 to 7.9% in 2014–2018, but similar to our previous study of breast cancer.^10^ There was a significant increase in fertility consultation rates across the study period (*p* for trend <0.001).

**Table 1.** Characteristics in Relationship to Fertility Consultation of Female, Lymphoma Patients in Ontario, Canada

Characteristic	Fertility consultation		*p*
	Yes,* n* = 71 (proportion of total, %)	No, *n* = 2017	
Type of cancer			
Hodgkin's lymphoma	44 (3.3)	1279	0.81
Non-Hodgkin's lymphoma	27 (3.5)	738	
Age at diagnosis			
15–29	49 (3.8)	1233	0.18
30–39	22 (2.7)	784	
Previous parity			
Nulliparous	63 (4.2)	1448	0.002
Parous	8 (1.4)	569	
Income quantile^a^			
1–3	33 (2.8)	1141–1146	0.2
4–5	38 (4.2)	870	
Deprivation score^a,b^			
1–3	54 (4.1)	1261	0.06
4–5	17 (2.2)	740	
Year of diagnosis			
2000–2006	7 (0.9)	772	<0.001
2007–2013	25 (3.1)	793	
2014–2018	39 (7.9)	452	
Time to chemotherapy			
≤6 weeks	38 (2.8)	1323	0.04
>6 weeks	33 (4.5)	694	

^a^ Both deprivation score and income quantile represent neighborhood scores not individualized to the oncology patient.

^b^ Deprivation score represents a combination score of socioeconomic factors as defined by 2011 Ontario Marginalization Index.

**Table 2.** Multivariable Analysis of Factors Influencing Fertility Consultations in Female, Adolescent and Young Adult Lymphoma Patients

Characteristic	Adjusted odds ratio (95% confidence interval)	*p*
Deprivation score^a,b^		
1–3 (reference)	1.0	0.04
4–5	0.55 (0.31–0.96)	
Parity		
Nulliparous (reference)	1.0	0.01
Parous	0.34 (0.16–0.73)	
Time to chemotherapy		
≤6 weeks (reference)	1.0	0.004
>6 weeks	2.03 (1.25–3.1)	
Year of diagnosis		
2000–2006 (reference)	1.0	< 0.001
2007–2013	3.48 (1.49–8.12)	
2014–2018	9.65 (4.26–21.9)	

^a^ Both deprivation score and income quantile represent neighborhood scores not individualized to the oncology patient.

^b^ Deprivation score represents a combination score of socioeconomic factors as defined by 2011 Ontario Marginalization Index.

A survey-based study investigating HM patients between 1957 and 2006 in Britain showed a slightly higher rate of 12% with improvement after 2000.^2^ However, fertility consultation in that study was publicly funded. In contrast, universal funding for fertility treatment began in Ontario in 2015. Referral rate in a private, American system for HMs, breast and gastrointestinal malignancy combined, was 5% between 1993 and 2007, similar to our study.^12^

Notably, we used fertility billing codes, which preclude referrals that were suggested, but not completed. While this code, ICD-9 code 628, is the only one for infertility and required for billing insured services in Ontario, its use as a substitute for fertility consultations has not been validated. Furthermore, it does not represent discussions had by other health care professionals, including oncologists, patient flow coordinators, and nursing staff. Together, these limitations may contribute to the low rates observed.

Fertility consults increased after the 2013 ASCO update (*p* < 0.001). This modest improvement is consistent with the literature that demonstrates knowledge gaps even after guidelines are released.^13^ Campbell et al. identified that 35% of pediatric oncologists surveyed read the 2013 guideline.^14^ In addition to guidelines, other mechanisms to promote oncofertility include the following: referral pathways, multidisciplinary teams, and provider education.^8,9^ One Canadian initiative is the establishment of the Canadian National Task Force on Adolescents and Young adults with Cancer, in 2008, which identified improving fertility outcomes in cancer survivors as a priority issue.^15^

Patient and socioeconomic factors may affect care. In this study, fertility consultations were not altered by patient age or cancer type. Several studies demonstrate that pediatric and older-AYA (30–39 year old) patients have reduced odds of referral than patients in their 20s.^8,10^ In both this study and the literature, patients with a history of childbirth were less likely to have a fertility consult than nulliparous women (odds ratio [OR] 0.34, 95% confidence interval [CI] 0.16–0.73).^10^

Since economic factors alter referral patterns, in Ontario, future research into the impacts of public funding is warranted.^16^ In Ontario, public funding of one cycle of *in vitro* fertilization for a female patient is covered under the Ontario Fertility Program, introduced in December 2015. Prior to this program, the consultation was publicly funded, but treatment was not. Thus, some patients may not have followed through with a consultation if they knew they could not afford fertility preservation.

Likewise, income quintile did not alter fertility consultation odds; however, more complex socioeconomic factors (e.g., education and employment) captured by a higher deprivation score decreased odds of consultation (OR 0.55; 95% CI 0.31–0.96). Being educated was similarly associated with increased referral trends in a review by Loren and Senapati,^9^ as well as heterosexuality and being Caucasian. This suggests social determinants of health influence oncofertility care.^17^

Fertility consultation was more likely if the time between diagnosis and chemotherapy was >6 weeks (OR 2.03, 95% CI 1.25–3.1). However, this result does not mean that fertility referrals delay treatment. The median time to chemotherapy was 5 weeks (IQR 3–8) and 6 weeks (IQR 4–9) for patients with and without a fertility consultation, respectively, thus demonstrating a modest delay (*p* = 0.02). Notably, we did not measure cancer stage, and therefore, cannot comment if urgency affects odds of referral. One possibility is that a less severe stage allows for more time for a referral. The literature and guidelines suggest early referrals and referral pathways reduce delays in treatment.^5,13^ Furthermore, more recent ovarian stimulation protocols allow for less delay.^7^

Several studies demonstrate that physician characteristics, including sex, attitudes, age, and specialty, affect referrals.^8,13^ Of the physicians who did refer, 60% were female, a higher proportion than female hematologists in Canada (<1/3 in the early 2000s increasing to 48% in 2018).^18^ Of the physicians who made a referral, 55% were older than 45. The most common specialty to refer was hematology (48%), which aligns with the target audience of the guidelines, followed by family physicians (20%) and medical oncology (11%).

Fertility consultation improved after the ASCO guidelines for female AYAs with lymphoma. By identifying referral trends, we can tailor future interventions toward demographics in need. Despite the modest improvement demonstrated in this study, oncofertility research demonstrates vast potential for improving care.
